# Supplementary material for: Long-Term Antifogging Coating Based on Black Phosphorus Hybrid Super-Hydrophilic Polymer Hetero-Network
Source: Nanomaterials (Basel). 2022 Dec 24;13(1):86. doi: 10.3390/nano13010086 (PMC9824178; doi:10.3390/nano13010086)
Supplement: Supplementary file 1 [file nanomaterials-13-00086-s001.zip › nanomaterials-2090463-supplementary.pdf]

nanomaterials

MDPI

Supplementary Materials

Instruments

The SEM images were acquired from the Zeiss SUPRATM 55 SAPPHIRE field-emission scanning electron microscope. The AFM images were acquired from the Bruker Icon atomic force microscope. FTIR spectra were collected in wavenumber range of 4000-400 cm^{-1} on a Thermo Nicolet IS5 instrument. Raman scattering was conducted on a Horiba Jobin-Yvon Lab Ram HR VIS high-resolution confocal Raman microscope equipped with a 633 nm laser. ^1H NMR spectroscopy was performed on the Bruker Advance DRX-300

spectrometer (Bruker, Germany) at 25 °C. The XPS spectra were obtained from a Thermo Escalab 250Xi spectrometer equipped with an X-ray source producing Al Kα radiation (1486.6 eV). The friction tests were completed by a MS-T3001 ball disc friction tester. Water contact angle tests were carried out by a SDP 260 contact angle/surface tension analyzer at room temperature.

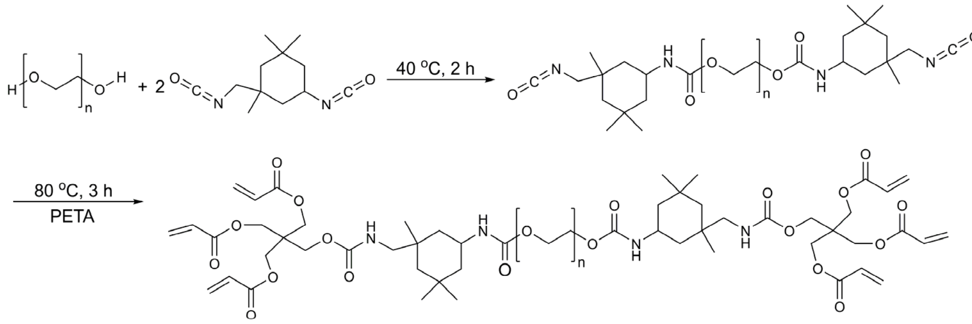

Figure S1. Schematic diagram of synthesis of polyurethane acrylate (PUA).

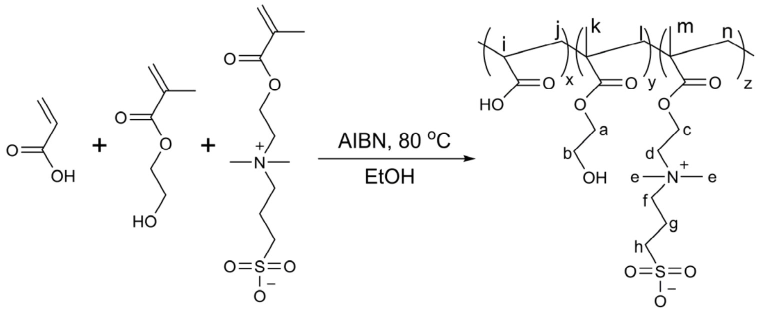

Figure S2. Schematic diagram of synthesis of P(AA-HEMA-SBMA) acrylic resin (PAHS).

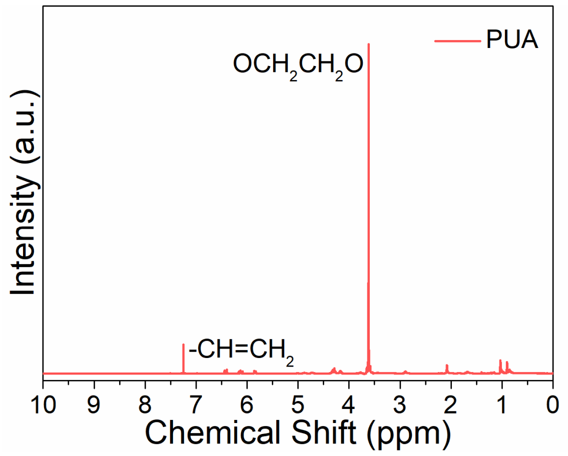

Figure S3. ^{1}H NMR spectrum of polyurethane acrylate (PUA): ^{1}H NMR (400 MHz, $CDCl_3$) δ_H 5.84-6.45 ($-CH{=}CH_2$), 4.27 ($-COOCH_2-$), 3.61 ($-OCH_2CH_2O-$), 0.90–2.08 ($-CH_3$, $-CH_2-$).

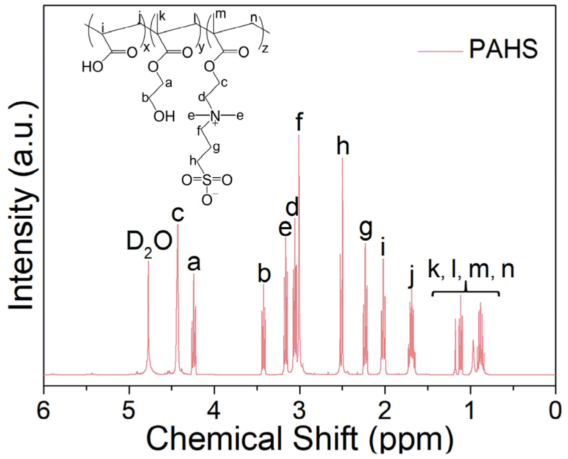

Figure S4. ^{1}H NMR spectrum of P(AA-HEMA-SBMA) acrylic resin (PAHS): ^{1}H NMR (400 MHz, D_2O) δ_H 4.48 (c), 4.24 (a), 3.42 (b), 3.21 (e), 3.05 (d), 3.02 (f), 2.51 (h), 2.23 (g), 2.02 (i), 1.68 (j) 0.88-1.17 (k, l, m, n).

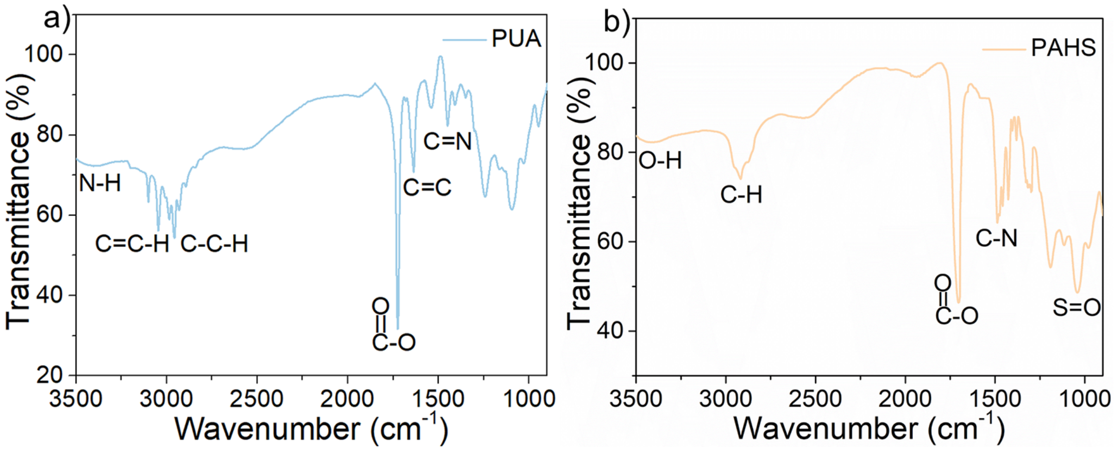

Figure S5. a) FTIR spectrum of polyurethane acrylate (PUA). b) FTIR spectrum of P (AA-HEMA-SBMA) acrylic resin (PAHS).

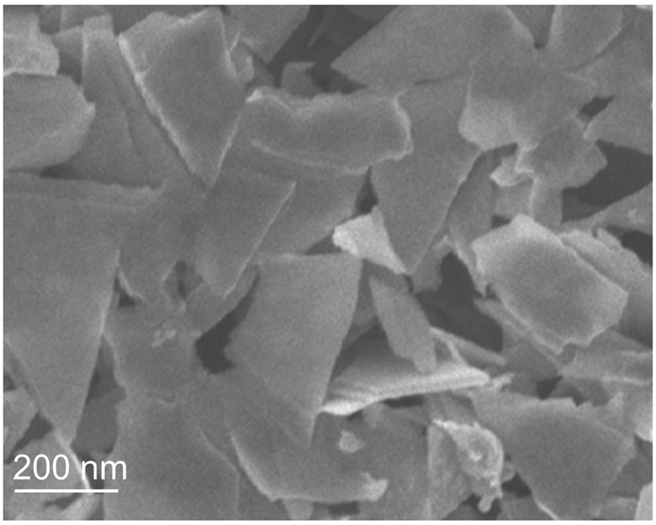

Figure S6. SEM image of black phosphorus nanosheets (BPs).

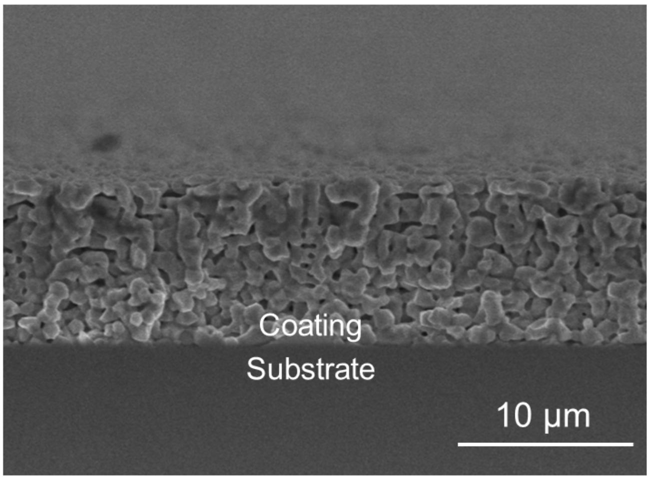

Figure S7. The cross-section SEM image of PUA/PAHS/BPs HN.

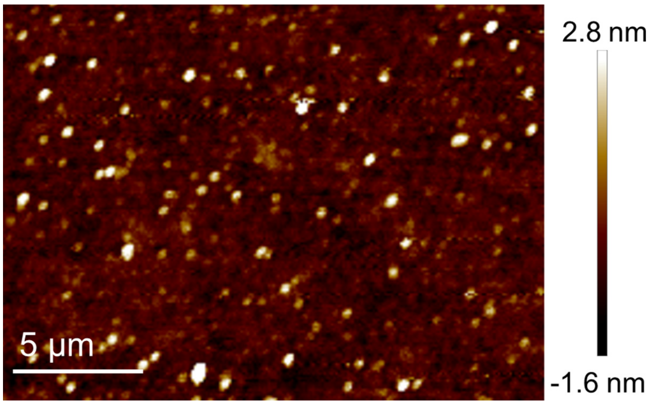

Figure S8. AFM image of PUA/PAHS/BPs HN.

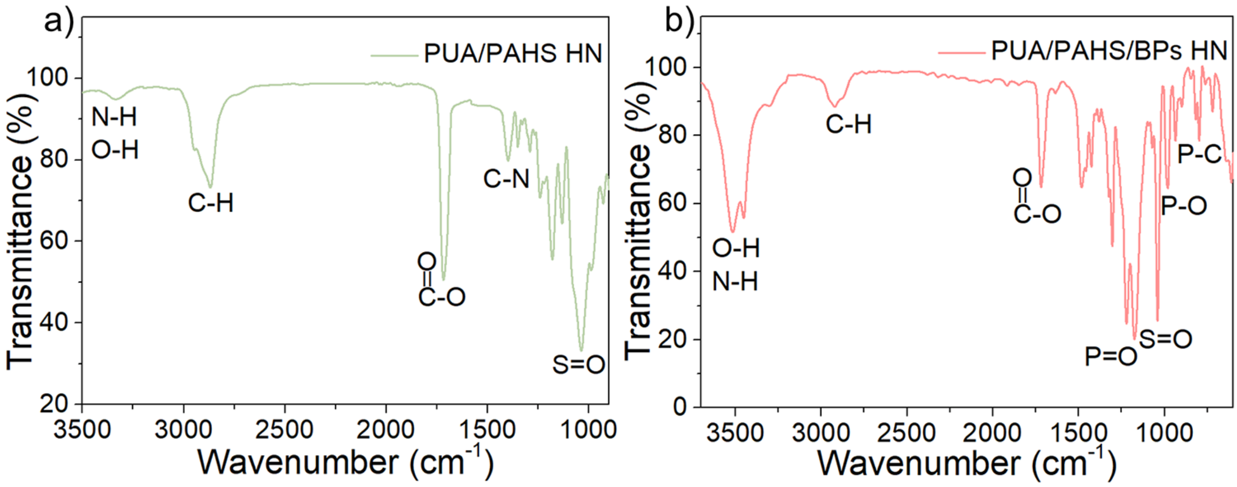

Figure S9. a) FTIR spectrum of PUA/PAHS HN. b) FTIR spectrum of PUA/PAHS/BPs HN.

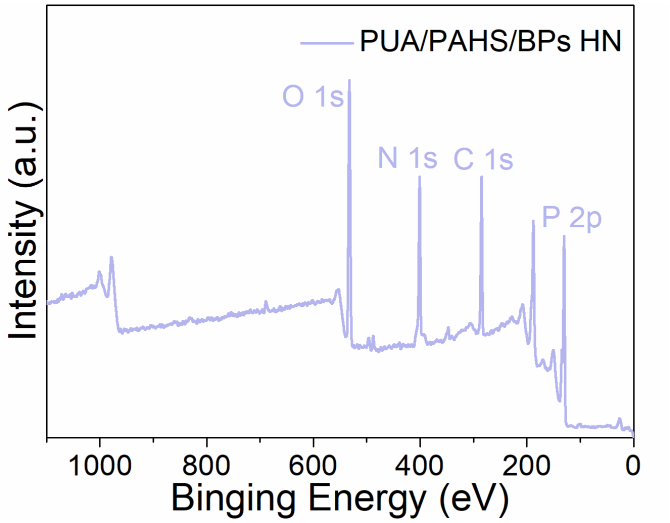

Figure S10. XPS spectrum of PUA/PAHS/BPs HN.

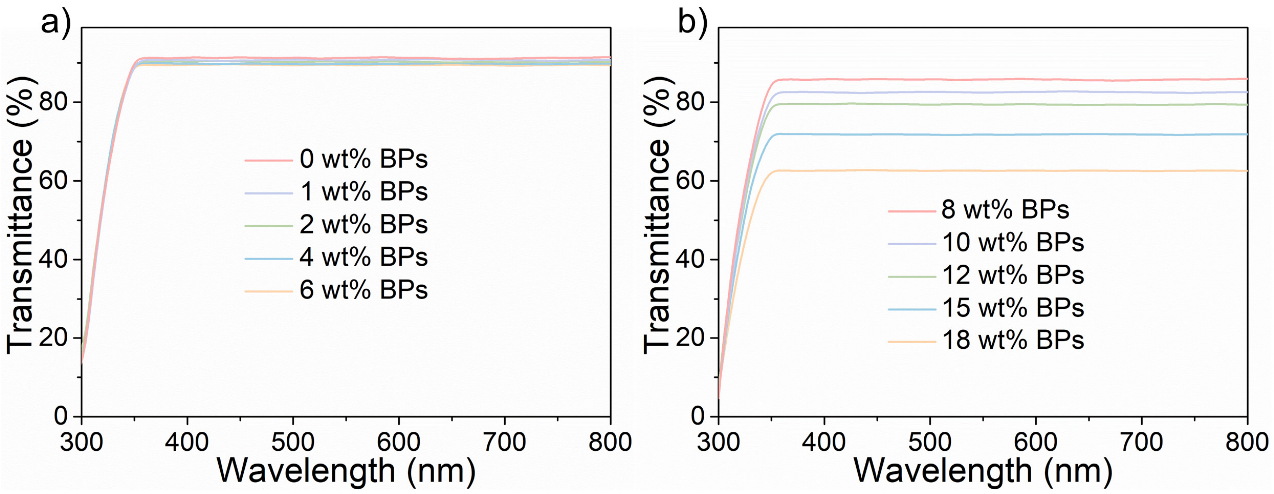

Figure S11. a) Transmission spectra of PUA/PAHS/BPs HN with 0 wt%, 1 wt%, 2 wt%, 4 wt% and 6 wt% BPs respectively. b) Transmission spectra of PUA/PAHS HN with 8 wt%, 10 wt%, 12 wt%, 15 wt%、18 wt% BPs respectively.

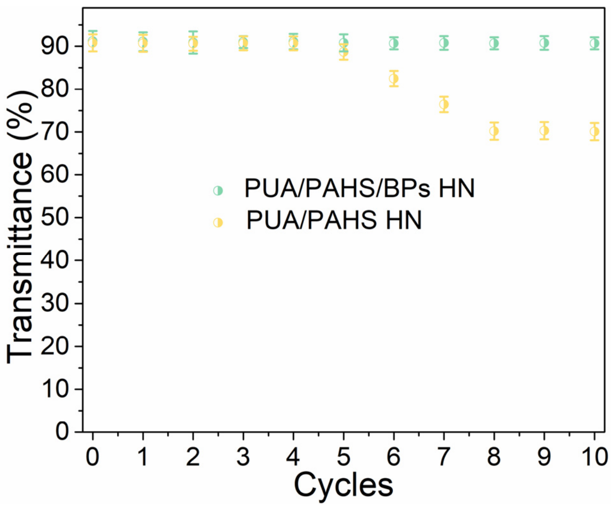

Figure S12. The average transmittance of a PMMA slide coated with PUA/PAHS/BPs HN during antifogging tests after high and low temperature cycles.
